# Stimulation of murine biliary cholesterol secretion by thyroid hormone is dependent on a functional ABCG5/G8 complex

**DOI:** 10.1002/hep.25861

**Published:** 2012-10-14

**Authors:** Ylva Bonde, Torsten Plösch, Folkert Kuipers, Bo Angelin, Mats Rudling

**Affiliations:** 1 Metabolism Unit, Center for Endocrinology, Metabolism, and Diabetes, Department of Medicine, Karolinska University Hospital HuddingeStockholm, Sweden; 2 Molecular Nutrition Unit, Department of Biosciences and Nutrition, Karolinska Institute, Karolinska University Hospital HuddingeStockholm, Sweden; 3 Center for Liver, Digestive, and Metabolic Diseases, Laboratory of Pediatrics, University Medical Center Groningen, University of GroningenGroningen, The Netherlands

## Abstract

Secretion of cholesterol into bile is important for the elimination of cholesterol from the body. Thyroid hormone (TH) increases biliary cholesterol secretion and hepatic gene expression of adenosine triphosphate (ATP)-binding cassette, subfamily G (WHITE), member 5 (ABCG5) and ATP-binding cassette, subfamily G (WHITE), member 8 (ABCG8), two half-transporters that act as a heterodimeric complex promoting sterol secretion. In addition, nuclear liver x receptor-alpha (LXRa), also regulated by TH, induces gene expression of ABCG5/G8. We here investigated if the TH-induced stimulation of biliary cholesterol secretion is mediated by the ABCG5/G8 complex *in vivo*, and if so, whether LXRa is involved. Mice homozygous for disruption of *Abcg5* (*Abcg5*^−/−^) or *Lxra* (*Lxra*^−/−^) and their wild-type counterparts were treated with triiodothyronine (T3) for 14 days and compared to untreated mice of corresponding genetic backgrounds. Bile was collected by gallbladder cannulation, and liver samples were analyzed for gene expression levels. Basal biliary cholesterol secretion in *Abcg5*^−/−^ mice was 72% lower than in *Abcg5*^+/+^ mice. T3 treatment increased cholesterol secretion 3.1-fold in *Abcg5*^+/+^ mice, whereas this response was severely blunted in *Abcg5*^−/−^ mice. In contrast, biliary cholesterol secretion in T3-treated *Lxra*^+/+^ and *Lxra*^−/−^ mice was increased 3.5- and 2.6-fold, respectively, and did not differ significantly. *Conclusions*: TH-induced secretion of cholesterol into bile is largely dependent on an intact ABCG5/G8 transporter complex, whereas LXRa is not critical for this effect. (HEPATOLOGY 2012;56:1828–1837)

High plasma total and low-density lipoprotein (LDL) cholesterol levels are linked to an enhanced risk of developing premature atherosclerosis. Thyroid hormone (TH) is an important regulator of cholesterol metabolism and hyperthyroidism is commonly associated with decreased—and hypothyroidism with increased—plasma cholesterol concentrations.^1–3^ TH is known to exert a number of beneficial effects on cholesterol and lipoprotein metabolism,^4^ and promising results have recently been reported from the clinical development of liver-selective TH analogs, such as eprotirome.^5^, ^6^ One of the mechanisms by which TH may lower plasma cholesterol is by an increased secretion of biliary cholesterol,^7^ a main route for elimination of cholesterol from the body.^8^

An important and presumably rate-limiting step in the process of biliary secretion of cholesterol is mediated by the half-transporters ATP-binding cassette, subfamily G (WHITE), member 5 (ABCG5) and ATP-binding cassette, subfamily G (WHITE), member 8 (ABCG8). By heterodimerization with each other, these structures form a functional complex that promotes the transport of cholesterol and plant sterols from liver cells into bile^9–11^ at the apical plasma membrane of hepatocytes.^10^, ^11^ Disruption of either one^12^, ^13^ or both^11^, ^14–16^ genes reduces biliary cholesterol concentration and secretion in mice. In contrast, induction of hepatic ABCG5/G8 gene expression is associated with increased biliary cholesterol concentration and secretion.^15–17^ In a previous study,^18^ biliary cholesterol secretion was strongly reduced in hypophysectomized rats as compared with intact animals, a finding associated with markedly reduced hepatic ABCG5/G8 gene expression. The administration of TH increased biliary cholesterol secretion and, concomitantly, hepatic ABCG5/G8 gene expression levels were increased. This suggests that TH-induced stimulation of biliary cholesterol secretion may be mediated by ABCG5/G8.

Hepatic gene expression of ABCG5/G8 is not always concurrent with biliary cholesterol secretion,^16^, ^19–21^ however, and there are indications that other pathways, independent of ABCG5/G8, promote cholesterol transfer into bile.^11^, ^22^, ^23^ Furthermore, it is unclear whether the stimulation of biliary cholesterol secretion is a direct effect of TH. It may well be mediated by nuclear liver x receptor-alpha (LXRa), the expression of which has recently been reported to be positively regulated by TH receptor-beta (TRb) in the mouse.^24^ LXRa regulates the transcription of several genes involved in cholesterol metabolism^25^ and the administration of the LXR agonist T0901317 to mice increases hepatic ABCG5/G8 gene expression and biliary cholesterol concentration and secretion.^15^, ^17^, ^26^ Thus, experimental evidence indicates that LXRa may mediate effects of TH on cholesterol metabolism.^27^

Here, we investigated whether the induction of biliary cholesterol secretion by TH is dependent on the ABCG5/G8 complex or if other mechanisms are involved. Furthermore, the question of if LXRa is important for the effect of TH on biliary cholesterol secretion was explored.

We present three novel findings: (1) Biliary cholesterol secretion induced by TH is predominantly excerted by ABCG5/G8; (2) this TH-induced biliary cholesterol secretion is independent of LXRa; and (3) a minor part of the TH-induced stimulation of biliary cholesterol secretion occurs independently of the ABCG5/G8 complex.

## Materials and Methods

### Animals and Treatments

Male mice (3–5 months of age) were used in the experiments. In the first experiment, mice homozygous for the disruption of the ABCG5 gene (*Abcg5*^−/−^) and their wild-type (WT) counterparts (*Abcg5*^+/+^) were divided into the following groups: *Abcg5*^+/+^ (n = 6); *Abcg5*^+/+^ T3 (n = 5); *Abcg5*^−/−^ (n = 5); and *Abcg5*^−/−^ T3 (n = 6). In the second experiment, mice homozygous for the disruption of the LXRa gene (*Lxra*^−/−^) and their WT counterparts (*Lxra*^+/+^) were divided into the following groups: *Lxra*^+/+^; *Lxra*^+/+^ T3; L*xra*^−/−^; and *Lxra*^−/−^ T3 (all groups: n = 7). For detailed descriptions of how knockout mice were generated, see previous reports.^13^, ^21^

Animals were housed in a temperature-controlled environment, with lights on from 6 a.m. to 6 p.m. They had free access to drinking water and mouse chow. Groups treated with T3 (*Abcg5*^+/+^ T3, *Abcg5*^−/−^ T3, *Lxra*^+/+^ T3, and *Lxra*^−/−^ T3) received drinking water supplemented with 0.5 μg of T3/mL (3,3′,5-triiodo-L-thyronine; Sigma-Aldrich, St Louis, MO) and 0.01% albumin (bovine serum albumin; Sigma-Aldrich). After 14 days of treatment, mice were anesthetized by an intraperitoneal injection of Hypnorm (fentanyl/fluanisone, 1 mL/kg) and diazepam (10 mg/kg). Bile was collected for 30 minutes from cannulated gallbladders, as previously described,^21^ and blood was collected by heart puncture at the end of the bile-collection period. After animals had been killed by cervical dislocation, livers were removed and immediately frozen in liquid nitrogen and stored at −80°C. All experimental procedures were approved by the Local Ethical Committee for Animal Experiments of the University of Groningen.

### RNA Isolation and Real-Time PCR Measurements

Total RNA was extracted from individual samples of liver and proximal small intestine using TRIzol Reagent (Invitrogen, Carlsbad, CA), according to the manufacturer's instructions. cDNA synthesis was performed using Omniscript reverse transcriptase (Qiagen, Hilden, Germany). Quantitative real-time PCR was performed with SYBRGreen PCR MasterMix on a 7500 Fast Real-Time PCR System, and primers were designed using Primer Express Software 2.0 (Applied Biosystems, Foster City, CA). Glyceraldehyde-3-phosphate dehydrogenase (*Gapdh*) and hypoxanthine guanine phosphoribosyl transferase (*Hprt*) were used as endogenous controls, and the comparative C_t_ method was used to quantify the results. The following primer sequences were used: *Gapdh* forward 5′-tgtgtccgtcgtggatctga-3′; *Gapdh* reverse 5′-cctgcttcaccaccttcttgat-3′; *Abcg5* forward 5′-aatgctgtg aatctgtttccca-3′; *Abcg5* reverse 5′-ccacttatgatacaggcca tcct-3′; *Abcg8* forward 5′-tccatcctcggagacacgat-3′; *Abcg8* reverse 5′-gctgatgccgatgacaatga-3′; *Lxra* forward 5′-gctct gctcattgccatcag-3′; *Lxra* reverse 5′-tgttgcagcctctctactt gga-3′; *Hprt* forward 5′-ggtgaaaaggacctctcgaagtg-3′; *Hprt* reverse 5′-atagtcaagggcatatccaacaaca-3′; *Cyp7a1* forward 5′-agcacctaaacaacctgccagtacta-3′; *Cyp7a1* reverse 5′-gtccggatattcaaggatgca-3′; *Hmgcr* forward 5′-tgattggagttggcaccat-3′; *Hmgcr* reverse 5′- tggccaacactga catgc-3′; *Ldlr* forward 5′-ggatggctatacctacccctcaa-3′; and *Ldlr* reverse 5′-cacatcgtcctccaggctg-3′.

### Assay of Biliary Cholesterol Concentration and Secretion

25 μL of bile was used for this assay. After Folch extraction, dried samples were hydrolyzed with 1 mL of 0.5 M KOH at 70°C for 90 min. Samples were extracted by the addition of 1 mL of H_2_O and 5 mL of hexane. After centrifugation at 3,000 rpm for 5 min, the upper phase was evaporated under nitrogen and silylated with pyridine/hexametyldisilazane/chlorotrimetylsilane (3:2:1, v/v/v) at 60°C for 30 min. After evaporation, the product was redissolved in hexane and analyzed using gas chromatography/mass spectrometry (GC/MS). D_7_-cholesterol was used as internal standard. Biliary cholesterol secretion was calculated for each individual by multiplying the cholesterol concentration by the volume of bile secreted per minute and per 100 g of body weight.

### Assay of Biliary Phospholipid Concentration and Secretion

Phospholipids were extracted from individual bile samples, as previously described.^28^ The concentration was subsequently determined as in Böttcher et al.^29^ Secretion of phospholipids was calculated for each individual by multiplying the concentration of phospholipids by the volume of bile secreted per minute and per 100 g of body weight.

### Assay of Biliary Bile Acid Concentration and Secretion

2 μL of bile were hydrolyzed with 0.5 mL of 5 M NaOH in 90% EtOH at 67°C for 90 min. Then, 0.5 mL of H_2_O and 3 mL of cyklohexane were added and samples were centrifuged at 2,000 rpm for 10 min before upper phase was removed. This was repeated once before acidification of samples with 200 μL of 6 M HCl. Ether was added to extract bile acids (BAs) and H_2_O was added to collected ether extracts, which were centrifuged at 2,000 rpm for 10 min before the upper phase was collected and evaporated under nitrogen at 60°C. Methylation was carried out at room temperature for 10 min by adding 400 μL of toluene, 100 μL of MeOH, and 25 μL of trimethylsilyldiazomethane, and samples were then dried under nitrogen at 60°C. Samples were silylated with pyridine/hexametyldisilazane/chlorotrimetylsilane (3:2:1, v/v/v) at 60°C for 30 min and thereafter dried under nitrogen, redissolved in hexane, and analyzed using GC/MS. D_4_-labeled BAs were used as internal standards. BA secretion was calculated for each individual by multiplying the sum of concentrations of specific BAs by the volume of bile secreted per minute and per 100 g of body weight.

### Statistical Analyses

Data show means ± standard error of the mean (SEM). The significance of differences between groups was tested by 1-way ANOVA, followed by post-hoc comparisons according to Tukey's test, using GraphPad Prism software (GraphPad Software Inc., San Diego, CA).

## Results

### Effects of T3 on Hepatic Gene Expression in *Abcg5*^+/+^ and *Abcg5*^−/−^ Mice

**Figure 1:**
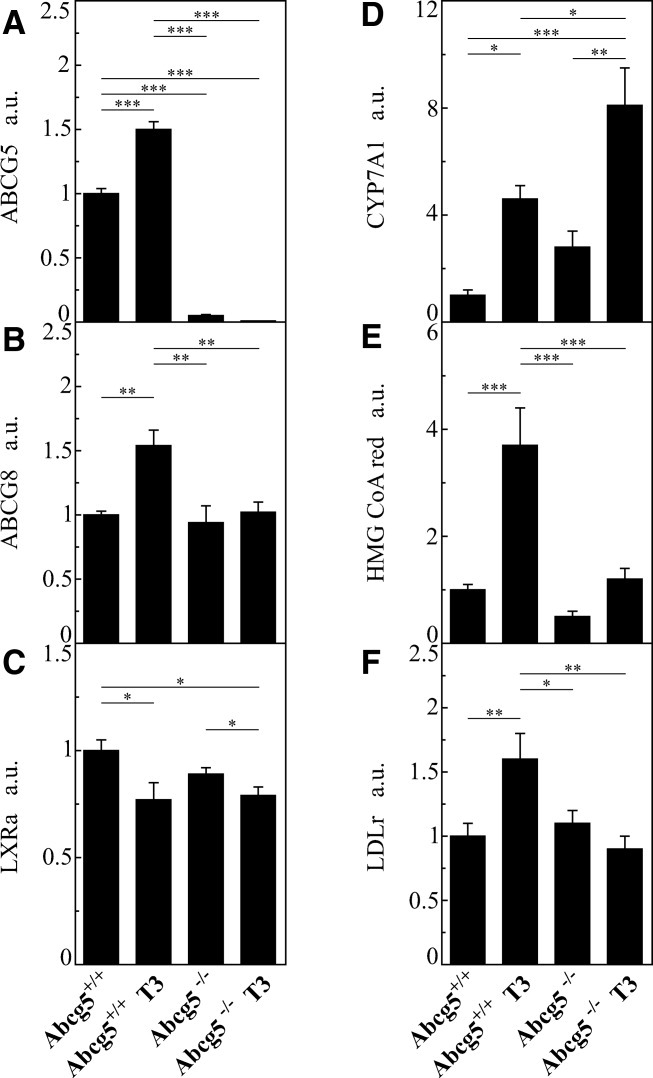
Effects of T3 treatment on hepatic gene expression of ABCG5 (A), ABCG8 (B), LXRa (C), CYP7A1 (D), HMG CoA red (E), and LDLr (F) in *Abcg5*^−/−^ mice and in their WT counterparts (*Abcg5*^+/+^). Number of animals (n) per group: *Abcg5*^+/+^ n = 6; *Abcg5*^+/+^ T3 n = 5; *Abcg5*^−/−^ n = 5; and *Abcg5*^−/−^ T3 n = 6. Data are presented as mean ± SEM. ***P < 0.001; **P < 0.01; *P < 0.05.

Hepatic ABCG5 and ABCG8 gene expressions were both increased 1.5-fold in T3-treated *Abcg5*^+/+^ mice (Fig. .1). ABCG8 gene expression was unaltered in *Abcg5*^−/−^ control and in T3-treated *Abcg5*^−/−^ mice. Hepatic LXRa gene expression was unaltered in *Abcg5*^−/−^ mice, while reduced in T3-treated *Abcg5*^+/+^ and *Abcg5*^−/−^ mice (by 23% and 10%, respectively), as compared to respective controls. Compared to untreated *Abcg5*^+/+^ mice, CYP7A1, hydroxymethylglutaryl coenzyme A reductase (HMG CoA red), and LDLr gene expressions were increased 4.6-, 3.7-, and 1.6-fold, respectively, in T3-treated *Abcg5*^+/+^ mice, whereas they were unaltered in untreated *Abcg5*^−/−^ mice. In T3-treated *Abcg5*^−/−^ mice, gene expressions of HMG CoA red and LDLr were unaltered, whereas CYP7A1 gene expression was 2.9-fold increased.

### Biliary Cholesterol, Phospholipids, BAs, and Bile Flow in T3-Treated *Abcg5*^+/+^ and *Abcg5*^−/−^ Mice

In *Abcg5*^−/−^ mice, biliary cholesterol and phospholipid concentrations were reduced by 75% and 46%, respectively (Table 1). In T3-treated *Abcg5*^+/+^ mice, biliary cholesterol and phospholipids were increased 1.8- and 1.3-fold, respectively. Compared to untreated *Abcg5*^−/−^ mice, cholesterol and phospholipids were unaltered in T3-treated *Abcg5*^−/−^ mice.

**Table 1:** Effects of T3 Treatment on Body Weight, Biliary Lipids, and Bile Flow in *Abcg5*^−/−^ Mice and in Their WT Counterparts (*Abcg5*^+/+^)

No. of Animals	*Abcg5^+/+^* (n = 6)	*Abcg5^++^* T3 (n = 5)	*Abcg5^−/−^* (n = 5)	*Abcg5^−/−^* T3 (n = 6)
Body weight, g	28 ± 1	31 ± 1	28 ± 1	32 ± 1
Cholesterol, nmol/mL	180 ± 10	320 ± 10*	50 ± 5*§	70 ± 10*§
Phospholipids, nmol/mL	6,060 ± 320	7,890 ± 420‡	3,300 ± 180*§	4,460 ± 400‡§
BAs, nmol/mL	48,000 ± 9,770	44,800 ± 8,530	22,100 ± 3,290	18,700 ± 1,110‡
Ratio cholesterol/phospholipid	0.03 ± 0.003	0.04 ± 0.002†	0.01 ± 0.000*§	0.02 ± 0.002*§
Ratio cholesterol/BA	0.004 ± 0.001	0.008 ± 0.001†	0.002 ± 0.000§	0.004 ± 0.001‖
Ratio phospholipid/BA	0.14 ± 0.02	0.20 ± 0.03	0.16 ± 0.03	0.24 ± 0.02‡
Bile flow, μL/min	2.0 ± 0.1	3.9 ± 0.3*	2.1 ± 0.2§	3.7 ± 0.3*¶

Data are presented as mean ± SEM.

* *P* < 0.001,

† *P* < 0.01, and

‡ *P*<0.05 versus *Abcg5*^+/+^.

§ *P* < 0.001 and

‖ *P* < 0.01 versus *Abcg5*^+/+^ T3.

¶ *P* < 0.001 versus *Abcg5*^−/−^.

The concentration of total BAs was unaltered in *Abcg5*^−/−^ mice. T3 treatment of *Abcg5*^+/+^ and *Abcg5*^−/−^ mice did not significantly change biliary BA concentration compared to respective controls. Both the C/PL ratio and C/BA ratio were increased (1.4- and 1.9- fold, respectively) in T3-treated *Abcg5*^+/+^ mice, whereas the PL/BA ratio was unaltered. In *Abcg5*^−/−^ mice, the C/PL ratio was decreased by 40%, and the C/BA and PL/BA ratios were unaltered. T3 treatment of *Abcg5*^−/−^ mice did not alter the ratios. Under basal conditions, bile flow was the same in *Abcg5*^+/+^ and *Abcg5*^−/−^ mice. T3 treatment increased bile flow to similar extents in *Abcg5*^+/+^ (1.9-fold) and in *Abcg5*^−/−^ (1.8-fold) mice.

### Effects of T3 on Biliary Composition of BAs in *Abcg5*^+/+^ and *Abcg5*^−/−^ mice

T3 treatment decreased the biliary proportion of deoxycholic acid (DCA) in *Abcg5*^+/+^ and *Abcg5*^−/−^ mice by 56% and 55%, respectively (Table 2). The proportion of cholic acid (CA) tended to be reduced in T3-treated animals. Chenodeoxycholic acid (CDCA) was increased 1.8-fold by T3 treatment in *Abcg5*^−/−^ mice, and there was a trend to an increased proportion of CDCA in the *Abcg5*^+/+^ mice (*P* = 0.05). Alpha-muricholic acid (α-MCA) was increased by T3 treatment in both *Abcg5*^+/+^ and *Abcg5*^−/−^ mice 2.1- and 3.4-fold, respectively. Biliary proportions of β-muricholic acid (β-MCA), ursodeoxycholic acid (UDCA), and litocholic acid (LCA) were unaltered.

**Table 2:** Effects of T3 Treatment on Biliary BA Composition in *Abcg5*^−/−^ Mice and in Their WT Counterparts (*Abcg5*^+/+^)

No. of Animals	*Abcg5^+/+^* (n = 6)	*Abcg5^++^* T3 (n = 5)	*Abcg5^−/−^* (n = 5)	*Abcg5^−/−^* T3 (n = 6)
CA				
nmol/mL	23,500 ± 3,700	20,100 ± 3,330	11,500 ± 2,150‡	7,540 ± 840†¶
% of total	51 ± 3	46 ± 2	51 ± 3	40 ± 3
CDCA				
nmol/mL	170 ± 10	260 ± 30‡	100 ± 10§	160 ± 20‖
% of total	0.4 ± 0.05	0.7 ± 0.09	0.5 ± 0.06	0.9 ± 0.08***
α-MCA				
nmol/mL	1,990 ± 410	4,220 ± 1,210	430 ± 70‖	1,190 ± 70¶
% of total	4.0 ± 0.4	9.0 ± 1.1*	2.0 ± 0.1§	6.0 ± 0.4‡¶#
β-MCA				
nmol/mL	21,300 ± 5,720	19,700 ± 4,400	9,420 ± 1,200	9,490 ± 800
% of total	42 ± 3	44 ± 3	44 ± 3	51 ± 3
DCA				
nmol/mL	510 ± 80	200 ± 10†	470 ± 70¶	180 ± 30†††
% of total	1.0 ± 0.1	0.5 ± 0.1‡	2.0 ± 0.1*§	1.0 ± 0.2#
UDCA				
nmol/mL	570 ± 100	350 ± 20	200 ± 30†	170 ± 20*
% of total	1.0 ± 0.2	1.0 ± 0.1	1.0 ± 0.1	1.0 ± 0.1
LCA				
nmol/mL	20 ± 0	20 ± 1	20 ± 0	20 ± 1
% of total	0.02 ± 0.02	0.02 ± 0.02	0.1 ± 0.00†‖	0.1 ± 0.00*‖

Data are presented as mean ± SEM.

* *P* < 0.001,

† *P* < 0.01, and

‡ *P* < 0.05 versus *Abcg5*^+/+^.

§ *P* < 0.001,

‖ *P* < 0.01, and

¶ *P* < 0.05 versus *Abcg5*^+/+^ T3.

# *P* < 0.001,

** *P* < 0.01, and

†† *P* < 0.05 versus *Abcg5*^−/−^.

### Importance of a Functional ABCG5/ABCG8 Complex for the Stimulation of Biliary Cholesterol Secretion by T3

**Figure 2:**
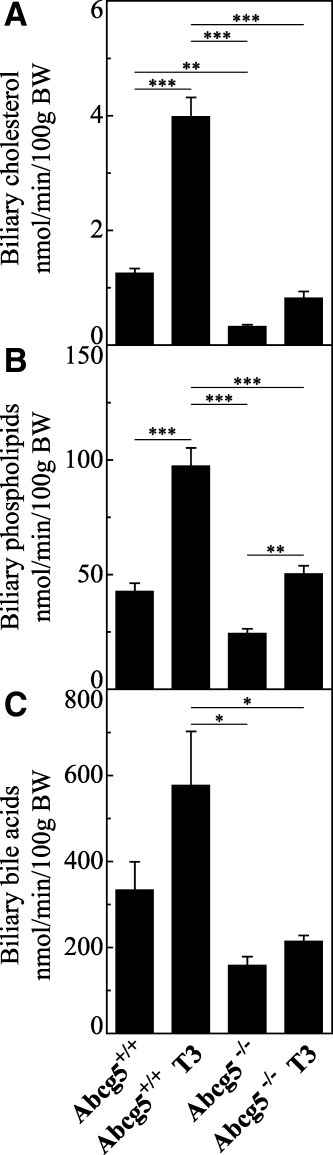
Effects of T3 treatment on secretion of biliary cholesterol (A), phospholipids (B), and BAs (C) in *Abcg5*^−/−^ mice and in their WT counterparts (*Abcg5*^+/+^). Number of animals (n) per group: *Abcg5*^+/+^ n = 6; *Abcg5*^+/+^ T3 n = 5; *Abcg5*^−/−^ n = 5; *Abcg5*^−/−^ T3 n = 6. Data are presented as mean ± SEM. ***P < 0.001; **P < 0.01; *P < 0.05.

Biliary cholesterol secretion was increased 3.1-fold in T3-treated *Abcg5*^+/+^ mice (Fig. .2). Basal secretion of biliary cholesterol in *Abcg5*^−/−^ mice was only 28% of that observed in untreated *Abcg5*^+/+^ mice. In T3-treated *Abcg5*^−/−^ mice, biliary cholesterol secretion was unaltered compared to *Abcg5*^−/−^ mice, and did not differ from that of untreated *Abcg5*^+/+^ mice. Biliary cholesterol secretion in T3-treated *Abcg5*^−/−^ mice was 79% lower than in T3-treated *Abcg5*^+/+^ mice. Biliary phospholipid secretion was unaltered in *Abcg5*^−/−^ mice. T3 treatment increased phospholipid secretion 2.3-fold in *Abcg5*^+/+^ mice and 2.1-fold in *Abcg5*^−/−^ mice, compared to respective controls. Total BA secretion was unaltered in *Abcg5*^−/−^ mice. T3 treatment of *Abcg5*^+/+^ and *Abcg5*^−/−^ mice tended to increase BA secretion, but the differences did not reach statistical significance.

### Effects of T3 on Hepatic Gene Expression in *Lxra*^+/+^ and *Lxra*^−/−^ Mice

**Figure 3:**
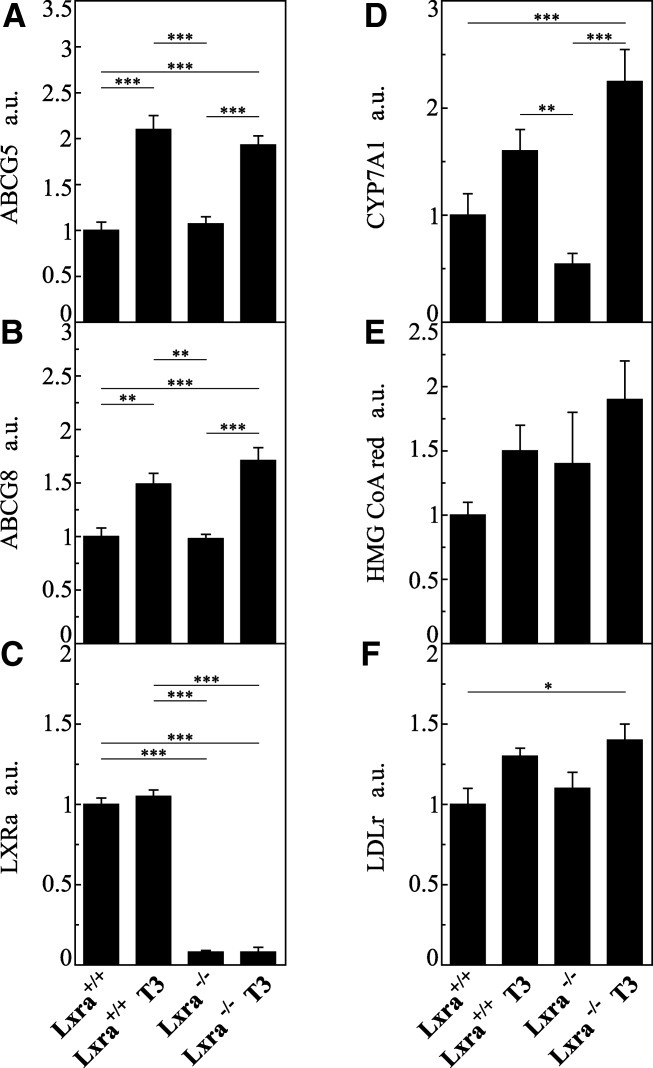
Effects of T3 treatment on hepatic gene expression of ABCG5 (A), ABCG8 (B), LXRa (C), CYP7A1 (D), HMG CoA red (E), and LDLr (F) in in *Lxra*^−/−^ mice and in their WT counterparts (*Lxra*^+/+^). Number of animals per group: n = 7. Data are presented as mean ± SEM. ***P < 0.001; **P < 0.01; *P < 0.05.

LXRa gene expression was unaltered in T3-treated *Lxra*^+/+^ mice, whereas ABCG5 and ABCG8 gene expression levels were both increased 2.1- and 1.5-fold, respectively (Fig. .3). Gene expressions of ABCG5 and ABCG8 were unaltered in *Lxra*^−/−^ mice, whereas they were increased in T3-treated *Lxra*^−/−^ mice (1.8- and 1.7-fold, respectively), as compared to *Lxra*^−/−^ mice. Gene expressions of ABCG5/G8 in proximal small intestine were unaltered (data not shown). Hepatic CYP7A1, HMG CoA red, and LDL receptor (LDLr) gene expressions were unaltered in *Lxra*^−/−^ mice and in T3-treated *Lxra*^+/+^ mice. In T3-treated *Lxra*^−/−^ mice, HMG CoA red and LDLr gene expressions were unaltered, whereas CYP7A1 gene expression was increased 4.1-fold, compared with untreated *Lxra*^−/−^ mice.

### Effects of T3 Treatment on Biliary Cholesterol, Phospholipids, BAs, and Bile Flow in *Lxra*^+/+^ and *Lxra*^−/−^ Mice

Biliary cholesterol and phospholipids were unaltered in *Lxra*^−/−^ mice, and T3 treatment of *Lxra*^+/+^ or *Lxra*^−/−^ mice did not alter biliary cholesterol or phospholipid concentrations, as compared to respective controls (Table 3). Further, the total concentration of biliary BAs did not differ between groups. C/PL and C/BA ratios were unaltered in T3-treated *Lxra*^+/+^ mice, whereas the PL/BA ratio was 1.4-fold increased. None of the ratios were altered in the *Lxra*^−/−^ mice. T3 treatment of *Lxra*^−/−^ mice increased both the C/BA and PL/BA ratio (1.6- and 1.4-fold, respectively), whereas the C/PL ratio was unaltered, as compared to untreated *Lxra*^−/−^ mice. Bile flow was unaltered in *Lxra*^−/−^ mice. However, T3 treatment increased bile flow by 2.4-fold in *Lxra*^+/+^ and 2.1-fold in *Lxra*^−/−^ mice.

**Table 3:** Effects of T3 Treatment on Body Weight, Biliary Lipids, and Bile Flow in *Lxra*^−/−^ Mice and in Their WT Counterparts (*Lxra*^+/+^)

No. of Animals	*Lxra^+/+^* (n = 7)	*Lxra^+/+^* T3 (n = 7)	*Lxra^−/−^* (n = 7)	*Lxra^−/−^* T3 (n = 7)
Body weight, g	34 ± 1	40 ± 1†	36 ± 1	38 ± 2
Cholesterol, nmol/mL	190 ± 30	320 ± 60	340 ± 80	470 ± 50‡
Phospholipids, nmol/mL	5,870 ± 610	6,710 ± 930	6,670 ± 710	7,610 ± 320
BAs, nmol/mL	49,200 ± 5,390	43,900 ± 9,560	53,400 ± 7,330	41,900 ± 3,480
Ratio cholesterol/phospholipid	0.03 ± 0.00	0.05 ± 0.00	0.05 ± 0.01	0.06 ± 0.05‡
Ratio cholesterol/BA	0.004 ± 0.001	0.008 ± 0.001	0.007 ± 0.001	0.011 ± 0.001*¶
Ratio phospholipid/BA	0.12 ± 0.01	0.17 ± 0.01‡	0.13 ± 0.01	0.19 ± 0.01†¶
Bile flow, μL/min	1.7 ± 0.1	4.1 ± 0.5*	1.7 ± 0.2§	3.5 ± 0.2*‖

Data are presented as mean ± SEM.

* *P* < 0.001,

† *P* < 0.01, and

‡ *P* < 0.05 versus *Lxra*^+/+^.

§ *P* < 0.001 versus *Lxra*^+/+^ T3.

‖ *P* < 0.001 and

¶ *P* < 0.05 versus *Lxra*^−/−^.

### Effects of T3 on Biliary Composition of BAs in *Lxra*^+/+^ and *Lxra*^−/−^ Mice

CDCA was increased by T3 treatment in *Lxra*^+/+^ and *Lxra*^−/−^ mice (2.0- and 1.7-fold, respectively) (Table 4). α-MCA was increased by T3 treatment in *Lxra*^+/+^ and *Lxra*^−/−^ mice (2.2- and 1.8-fold, respectively). There was a tendency toward decreased biliary proportions of DCA and CA in T3-treated *Lxra*^+/+^ and *Lxra*^−/−^ mice, whereas the biliary proportions of β-MCA, UDCA, and LCA were unaltered.

**Table 4:** Effects of T3 Treatment on Biliary BA Composition in *Lxra*^−/−^ Mice and in Their WT Counterparts (*Lxra*^+/+^)

No. of Animals	*Lxra^+/+^* (n =7)	*Lxra^+/+^* T3 (n =7)	*Lxra^−/−^* (n =7)	*Lxra^−/−^* T3 (n =7)
CA				
nmol/mL	21,900 ± 3,320	18,900 ± 4,170	28,300 ± 4,700	19,300 ± 2,200
% of total	44 ± 4	43 ± 3	51 ± 3	45 ± 2
CDCA						
nmol/mL	670 ± 50	1,120 ± 180‡	730 ± 80	1.050 ± 80
% of total	1.0 ± 0.1	3.0 ± 0.4†	2.0 ± 0.2‖	3.0 ± 0.3‡††
α-MCA				
nmol/mL	2,860 ± 390	5,810 ± 1,560	3,970 ± 790	5,200 ± 370
% of total	6.0 ± 0.5	13.0 ± 0.9*	7.0 ± 0.6§	13.0 ± 0.9*#
β-MCA				
nmol/mL	21,400 ± 2,610	16,300 ± 3,610	18,100 ± 2,930	14,900 ± 1,210
% of total	43 ± 3	37 ± 2	34 ± 4	36 ± 2
DCA				
nmol/mL	1,320 ± 130	710 ± 110‡	1,370 ± 200¶	630 ± 70†**
% of total	3.0 ± 0.4	2.0 ± 0.2	3.0 ± 0.7	2.0 ± 0.2
UDCA				
nmol/mL	1,000 ± 90	1,060 ± 220	910 ± 110	830 ± 30
% of total	2.0 ± 0.1	2.0 ± 0.1	2.0 ± 0.1	2.0 ± 0.1
LCA				
nmol/mL	60 ± 2	60 ± 3	60 ± 2	50 ± 1
% of total	0.1 ± 0.01	0.2 ± 0.02	0.1 ± 0.03	0.1 ± 0.02

Data are presented as mean ± SEM.

* *P* < 0.001,

† *P* < 0.01, and

‡ *P* < 0.05 versus *Lxra*^+/+^.

§ *P* < 0.001,

‖ *P* < 0.01, and

¶ *P* < 0.05 versus *Lxra*^+/+^ T3.

# *P* < 0.001,

** *P* < 0.01, and

†† *P* < 0.05 versus *Lxra*^−/−^.

### Biliary Cholesterol Secretion Is Induced by T3 independent of *Lxra*

**Figure 4:**
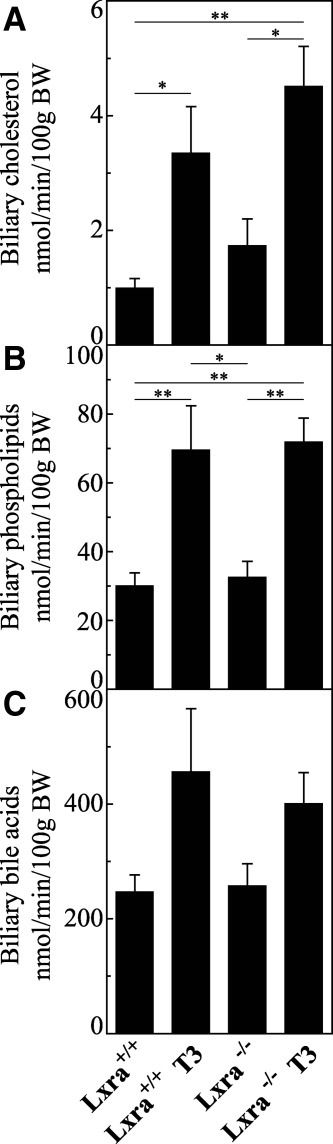
Effects of T3 treatment on secretion of biliary cholesterol (A), phospholipids (B), and BAs (C) in *Lxra*^−/−^ mice and in their WT counterparts (*Lxra*^+/+^). Number of animals per group: n = 7. Data are presented as mean ± SEM. **P < 0.01; *P < 0.05.

Biliary cholesterol secretion was similar in untreated *Lxra*^+/+^ and *Lxra*^−/−^ mice (Fig. .4). In response to T3 treatment, it increased 3.5-fold in *Lxra*^+/+^ and 2.6-fold in *Lxra*^−/−^ mice, to similar levels. Phospholipid secretion was unchanged in *Lxra*^−/−^ mice. In T3-treated *Lxra*^+/+^ mice and *Lxra*^−/−^ mice, phospholipid secretion increased 2.3- and 2.2-fold, compared to respective controls. Secretion of total BAs was unchanged in the groups, although there was a trend to an increased secretion in T3-treated mice.

## Discussion

TH exerts a number of important regulatory effects on cholesterol, lipid, and lipoprotein metabolism.^4^ These include stimulation of hepatic lipase activity, induction of hepatic LDL receptors, promotion of cholesterol breakdown to BAs, and cholesterol excretion into bile. Furthermore, there is evidence that TH may promote reverse cholesterol transport through stimulation of high-density lipoprotein (HDL) clearance.^4^, ^30^ Many of the positive actions of TH in lipid metabolism are constrained to the liver, and the recent demonstration of the possibility to achieve pronounced lipid-lowering effects in humans by selectively stimulating TRb in the liver has revitalized the interest for understanding the molecular effects of TH.^4–6^ We here explored by which mechanisms TH exerts its powerful effects on biliary cholesterol secretion by specifically analyzing the role of the ABCG5/G8 half-transporter complex in mice. This complex has been shown to be of major importance for sterol excretion into bile, but there are also data indicating that ABCG5/G8-independent mechanisms may promote cholesterol secretion. First, biliary cholesterol secretion/concentration is not completely abolished in single^12^, ^13^ and double^11^, ^14–16^ ABCG5/G8 knockout models. Second, hepatic overexpression of scavenger receptor class B, member 1 (SR-BI) in *Abcg5*^−/−^ mice can restore their initially decreased biliary cholesterol secretion to WT levels.^23^ And third, since transintestinal cholesterol efflux occurs in *Abcg5*^−/−^ 22 and *Abcg8*^−/−^31 mice via additional pathways not yet defined, such mechanisms may operate also in the liver.

To determine to which extent the strong stimulation of biliary cholesterol secretion induced by TH is mediated by the ABCG5/G8 complex, we treated *Abcg5*^−/−^ and WT mice of the same genetic background (*Abcg5*^+/+^) with T3. In line with previous results,^18^ TH treatment increased hepatic gene expression of ABCG5/G8 in *Abcg5*^+/+^ mice, but failed to increase ABCG8 gene expression in *Abcg5*^−/−^ mice. This lack of response may be the result of a disruption in a regulatory region of *Abcg8* caused in the procedure of disrupting *Abcg5*. The ABCG5 and ABCG8 genes are orientated in a head-to-head manner in the genome within 400 base pairs of each other. This implies that putative binding sites for transcription factors for one gene may be positioned within the opposite gene. Therefore, the insertion of the LacZ/Neo cassette used to disrupt the ABCG5 gene has been shown to also indirectly influence the expression of the other gene (ABCG8).^13^

Biliary cholesterol secretion was strongly reduced in *Abcg5*^−/−^ mice, to only 28% of that in *Abcg5*^+/+^ mice. T3 treatment increased biliary cholesterol secretion 3.1-fold in *Abcg5*^+/+^ mice, whereas in *Abcg5*^−/−^ mice, this response was blunted. These results demonstrate that stimulation of biliary secretion of cholesterol by T3 treatment of mice is largely dependent on an intact ABCG5/G8 complex. However, T3 treatment restored the low biliary secretion of cholesterol in *Abcg5*^−/−^ mice up to the basal rate observed in *Abcg5*^+/+^ mice. This suggests that, although a functional ABCG5/G8 complex is required for the major stimulation of biliary cholesterol secretion by T3, there is also an additional, ABCG5/G8-independent, mechanism.

The increased secretion in *Abcg5*^−/−^ mice occurred simultaneously with a T3-induced doubled flow rate of bile, regardless of the genetic background of the animals. Thus, one explanation for the non-ABCG5/G8 driven cholesterol secretion could be that it reflects the combined results of simple diffusion of cholesterol and the biliary capacity to bind cholesterol. The T3-induced flow rate of bile would then modulate the total output of diffusible lipophilic compounds such as cholesterol and phospholipids, as observed, and may in turn be related to circulatory effects exerted by the hormone. In addition to the markedly (3-fold) increased secretion of cholesterol, gene expression of the rate-limiting enzyme in BA synthesis, cholesterol 7α-hydroxylase (cytochrome P450 [CYP]7A1), was 4.6-fold increased by T3 treatment in *Abcg5*^+/+^ mice. These changes were accompanied with increased gene expression levels of the LDLr and HMG CoA red, the rate-limiting enzyme in cholesterol synthesis (2- and 4-fold, respectively), suggesting that the increased hepatic turnover of cholesterol is balanced by an increased *de novo* synthesis of cholesterol and by an increased uptake of cholesterol from the circulation. Consistent with previous results,^13^ the concentration of total BAs in bile was unchanged in *Abcg5*^−/−^ mice. In spite of an increased bile flow rate, and in contrast to the effect on the secretion of cholesterol, the secretion of total BAs was unaltered by T3 treatment. T3 treatment decreased the proportion of DCA and CA, whereas the proportions of CDCA and α-MCA increased. These results are in line with the concept that TH suppresses the BA synthetic enzyme, sterol 12-α-hydroxylase (CYP8B1), as has previously been shown.^32–34^

Activation of LXR by selective agonists has similar effects on hepatic ABCG5/G8 gene expression levels and biliary cholesterol secretion as TH.^15^, ^17^ It has been reported that LXRa is positively regulated at the transcriptional level by TRb.^24^ We therefore investigated the role of LXRa in the TH-induced stimulation of biliary cholesterol secretion. For this purpose, *Lxra*^−/−^ and *Lxra*^+/+^ mice with the same genetic background were treated with T3. Biliary cholesterol secretion rates did not differ between T3-treated *Lxra*^+/+^ and *Lxra*^−/−^ mice. These results clearly indicate that the stimulation of biliary cholesterol secretion in response to T3 is independent of LXRa. Mean levels of CYP7A1, HMG CoA red, and LDLr gene expressions were higher in T3-treated *Lxra*^+/+^ mice, compared to the controls. However, as opposed to the response in T3-treated *Abcg5*^+/+^ mice, mean levels were not statistically significantly different.

Because LXRa agonists have been shown to possess adverse side effects,^35–37^ the apparent absence of LXRa involvement in TH-induced responses on biliary cholesterol is promising from a therapeutic point of view, since available novel thyromimetics, such as eprotirome, should thus not be expected to present such side effects.

In conclusion, we have demonstrated that the ability of TH to stimulate the secretion of cholesterol into bile is largely mediated by the ABCG5/G8 complex, whereas LXRa does not seem to be of importance for this effect.

## Abbreviations

ABCG5: ATP-binding cassette, subfamily G (WHITE), member 5
ABCG8: ATP-binding cassette, subfamily G (WHITE), member 8
α-MCA: alpha-muricholic acid
ATP: adenosine triphosphate
BA: bile acid
β-MCA: beta-muricholic acid
CA: cholic acid
CDCA: chenodeoxycholic acid
CYP: cytochrome P450
DCA: deoxycholic acid
Gapdh: glyceraldehyde-3-phosphate dehydrogenase
GC: gas chromatography
HDL: high-density lipoprotein
HMG CoA red: hydroxymethylglutaryl coenzyme A reductase
Hprt: hypoxanthine guanine phosphoribosyl transferase
LCA: litocholic acid
LDL: low-density lipoprotein
LDLr: LDL receptor
LXRa: liver x receptor alpha
MS: mass spectrometry
T3: triiodothyronine
TH: thyroid hormone
TRb: thyroid hormone receptor beta
UDCA: ursodeoxycholic acid
WT: wild type
